# Improved renal function in neurofibromatosis type 1 patients

**DOI:** 10.1002/ski2.119

**Published:** 2022-05-02

**Authors:** Ken‐ichi Yasuda, Yoshimasa Nobeyama, Akihiko Asahina

**Affiliations:** ^1^ Dermatology Jikei University School of Medicine Minato‐ku Tokyo Japan

## Abstract

Neurofibromatosis type 1 (NF1), or von Recklinghausen disease, is an autosomal dominant disease that presents with various symptoms, including café‐au‐lait spots and neurofibromas. NF1 patients occasionally suffer from renal artery vasculopathy, which impairs renal function, while results of a previous report suggested that male NF1 patients have a low creatinine level in peripheral blood. The assessment of renal function in NF1 patients remains inadequate. In this study, renal function in NF1 was assessed. We recruited 308 patients consisting of 149 NF1 patients (77 males and 72 females) and 159 control patients (102 males and 57 females). Creatinine, blood urea nitrogen and haemoglobin A1c in peripheral blood as well as protein, occult blood and sugar in urine were examined. In addition, the estimated glomerular filtration rate was calculated. The mean age and body mass index did not differ significantly between the NF1 patients and controls for both sexes. For both sexes, i) the mean creatinine value was significantly lower in the NF1 patients than in the controls; ii) the mean blood urea nitrogen value did not differ significantly between the NF1 patients and controls; iii) the mean blood urea nitrogen‐to‐creatinine ratio was significantly higher in the NF1 patients than in the controls; iv) the mean estimated glomerular filtration rate was significantly higher in the NF1 patients than in the controls; and v) the mean haemoglobin A1c value was significantly lower in the NF1 patients than in the controls. In conclusion, NF1 patients may have improved renal function. The clinical significances should be further examined.

1

**What is already known about this topic?**

- Previous studies reported that neurofibromatosis type 1 negatively affects renal function through high blood pressure mostly caused by renal artery stenosis and pheochromocytoma/paraganglioma in children.  In contrast, there has been a report that neurofibromatosis type 1 positively affects renal function.  Thus, it remains unclear whether patients with neurofibromatosis type 1 have favorable renal function.

**What does this study add?**

- This study assessed renal function in patients with neurofibromatosis type 1 through the comparison between the patient groups with and without neurofibromatosis type 1.  For both sexes, the mean creatinine value in the peripheral blood and mean estimated glomerular filtration rate were significantly lower and higher, respectively, in the patients with neurofibromatosis type 1 than in the controls.  This study strongly suggests that patients with neurofibromatosis type 1 may have improved renal function.

## 2 INTRODUCTION

Neurofibromatosis type 1 (NF1), or von Recklinghausen disease, is an autosomal dominant disease that presents with various symptoms and signs, including café‐au‐lait spots, axillary freckling, cutaneous neurofibromas, and plexiform neurofibromas.^1^ The disease is mainly caused by mutation of the *NF1* gene encoding neurofibromin, which negatively regulates the RAS/MAPK pathway.

Previous studies reported that NF1 negatively affects renal function through high blood pressure. The incidence of hypertension in NF1 patients is approximately 16%,^2^, ^3^ and it is mostly caused by renal artery stenosis^4^, ^5^ and pheochromocytoma/paraganglioma^6^, ^7^ in children. Triantafyllidi et al. reported a case in which renal artery aneurysms were the cause of severe hypertension.^8^ Ueda et al. reported a paediatric case of hypertension that persisted despite successful dilation of a stenotic renal artery.^9^ Thus, the renal function in NF1 patients is sometimes impaired by vasculopathy, which is the most common cause of death after malignancies in NF1 patients.^3^, ^10^ Based on these data, the renal function of NF1 patients appears to be poor due to complicated vasculopathy. However, there has been a report that NF1 positively affects renal function; Koga et al. recently reported that male NF1 patients have a significantly lower level of creatinine in peripheral blood.^11^ Nonetheless, it remains unclear whether NF1 patients have favourable renal function, because i) the creatinine level in peripheral blood is easily affected by age, and the median age was lower by 2.5 years in the NF1 patient groups than the controls in that study, and ii) renal function is known to be affected by microangiopathy due to diabetes mellitus, and the median body mass index (BMI), which is closely associated with diabetes mellitus, was significantly lower in the male NF1 patients than in the controls in that study. As such, a study to more precisely evaluate the renal function of NF1 patients is needed.

In this study, we analysed and compared the renal function of 149 NF1 patients and 159 control patients whose age and BMI did not differ significantly to better understand the renal function in NF1 patients.

## 3 PATIENTS AND METHODS

### 3.1 Patients and assessments

The ethics committee of The Jikei University School of Medicine, Tokyo, Japan, approved the study protocol, and informed consent was obtained in the form of opt‐out for all patients.

We recruited 149 NF1 patients [77 males and 72 females] and 159 non‐NF1 patients (102 males and 57 females; these included patients suffering from lipoma [31 males and 20 females], atheroma [59 males and 24 females], and melanocytic nevus [12 males and 13 females]) who met the following criteria: i) referral to The Jikei University School of Medicine; ii) for NF1 patients, fulfilment of the NF1 diagnostic criteria of the National Institutes of Health Consensus Development Conference in 1987^12^; iii) surgical excision for the neurofibroma, lipoma, atheroma, or melanocytic nevus; iv) results available from blood and urine testing for preoperative checks within 6 weeks before the operation; and v) no acute diseases at the time of the preoperative check or the operation. The clinical data of each patient are provided in Table S1 and S2.

Comorbidities, including hypertension, renal artery stenosis, pheochromocytoma and paraganglioma, listed in medical records were examined. A diagnosis of scoliosis was confirmed by measuring Cobb's angle on anterior‐posterior radiographic images of the vertebral column. Hypertension was defined as a systolic blood pressure ≥140 mmHg and/or a diastolic blood pressure ≥90 mmHg. Scoliosis was defined as a spinal condition with Cobb's angle ≥11°.

Biochemical blood analysis and qualitative urinalysis were performed with an automatic analyser LABOSPECT 008 α (Hitachi) and an UTION MAX AX‐4060 (ARKRAY, Tokyo, Japan), respectively. The normal creatinine ranges were defined as 0.65–1.07 mg/dl and 0.46–0.79 mg/dl in males and females, respectively. The normal blood urea nitrogen (BUN) and haemoglobin A1c (HbA1c) ranges were defined as 8.0–20.0 mg/dl and 4.6%–6.2%, respectively, for both sexes. The creatinine‐based estimated glomerular filtration rate (eGFR) was calculated using the following formula^13^:

$$eGFR\;\left(mL/min/1.73\;m^{2}\right)\;=\;194\times serum\;creatinine^{-1.094}\times age^{-0.287}\left(for\;male\right)$$

$$eGFR\;\left(mL/min/1.73\;m^{2}\right)\;=\;194\times serum\;creatinine^{-1.094}\times age^{-0.287}\times 0.739\;\left(for\;female\right)$$

### 3.2 Statistical analysis

Statistical analyses were performed with the commercially available software SPSS version 22 (SPSS). The Mann‐Whitney *U* test was used to examine quantitative differences between the NF1 patients and control patients. The Chi‐squared test was used to examine qualitative differences between the NF1 patients and control patients. A *p* < 0.05 was considered to be statistically significant.

## 4 RESULTS

### 4.1 Assessment of control data

The mean age and BMI did not differ significantly between the NF1 patients and non‐NF1 patients with lipoma, atheroma, or melanocytic nevus in both sexes (Table 1, Table S1 and S2). The mean creatinine values were 0.92 and 0.66 mg/dl, the mean BUN values were 15.8 and 13.2 mg/dl, and the mean HbA1c values were 5.84% and 5.53% in the male and female non‐NF1 patients, respectively. These values were within the normal ranges by wide margins. Based on this, we regarded the non‐NF1 patients as normal controls.

**TABLE 1:** Comparison of clinical data between NF1 patients and controls

	Mean value ± standard deviation			
	Sex	NF1	Control	*p*‐value
Age (years)	Male	51.9 ± 10.8	52.9 ± 16.1	0.084
	Female	51.4 ± 10.0	50.3 ± 15.7	0.632
BMI (kg/m^2^)	Male	22.5 ± 4.5	23.9 ± 3.8	0.130
	Female	21.5 ± 3.2	22.1 ± 3.9	0.818
Creatinine (mg/dl)	Male	0.82 ± 0.13	0.92 ± 0.48	<0.001
	Female	0.60 ± 0.12	0.66 ± 0.11	0.001
BUN (mg/dl)	Male	15.9 ± 8.2	15.2 ± 4.1	0.582
	Female	13.3 ± 3.6	13.2 ± 4.4	0.851
BUN/creatinine	Male	19.5 ± 8.8	17.4 ± 5.1	0.001
	Female	22.9 ± 8.1	20.3 ± 7.2	0.035
eGFR (ml/min/1.73 m^2^)	Male	80.7 ± 15.2	77.5 ± 31.3	<0.001
	Female	84.9 ± 18.4	79.3 ± 29.4	0.004
HbA1c (%)	Male	5.58 ± 0.61	5.84 ± 0.93	0.001
	Female	5.36 ± 0.33	5.53 ± 0.56	0.031

Abbreviations: BMI, body mass index; BUN, blood urea nitrogen; eGFR, estimated glomerular filtration rate.

### 4.2 Creatinine, BUN, eGFR, and HbA1c in NF1 patients and controls

The creatinine level, BUN level, BUN‐to‐creatinine ratio, and eGFR were compared between the NF1 patients and controls (Table 1). The mean creatinine values were 0.79 and 0.92 mg/dl in male, and 0.60 and 0.66 mg/dl in female NF1 patients and controls, respectively. The mean BUN values were 16.0 and 15.2 mg/dl in male, and 13.3 and 13.2 mg/dl in female NF1 patients and controls, respectively. The mean BUN‐to‐creatinine ratios were 20.5 and 17.2 in male, and 22.9 and 20.3 in female NF1 patients and controls, respectively. The mean eGFRs were 84.6 and 77.5 ml/min/1.73 m^2^ in male, and 84.9 and 79.3 ml/min/1.73 m^2^ in female NF1 patients and controls, respectively. The mean HbA1c values were 5.58% and 5.84% in male, and 5.36% and 5.53% in female NF1 patients and controls, respectively.

In both sexes, statistical analyses revealed that: i) the mean creatinine value was significantly lower in the NF1 patients than in the controls; ii) the mean BUN value did not differ significantly between the NF1 patients and controls; iii) the mean BUN‐to‐creatinine ratio was significantly higher in the NF1 patients than in the controls; iv) the mean eGFR was significantly higher in the NF1 patients than in the controls; and v) the mean HbA1c value was significantly lower in the NF1 patients than in the controls (Table 1).

### 4.3 Urinalysis data of NF1 patients and controls

Urinalysis data were available for 73 male NF1 patients, 94 male control patients, 69 female NF1 patients, and 48 female control patients. The ratios of patients with urine protein were 28/73 (38.4%) and 39/94 (41.5%) among the male NF1 patients and controls, respectively, and 21/69 (30.4%) and 12/48 (25.0%) among the female NF1 patients and controls, respectively (Table 2). Similarly, the ratios of patients with urine occult blood were 8/73 (11.0%) and 16/94 (17.0%) among the male NF1 patients and controls, respectively, and 19/69 (27.5%) and 17/48 (35.4%) among the female NF1 patients and controls, respectively. The ratios of patients with urine sugar were 3/73 (4.1%) and 5/94 (5.3%) among the male NF1 patients and controls, respectively, and 1/69 (1.4%) and 0/48 (0.0%) among the female NF1 patients and controls, respectively.

**TABLE 2:** Rates of positive urinalysis in NF1 and control patients

	Rate in NF1 patients	Rate in controls	*p*‐value
Male patients			
Urine protein	28/73 (38.4%)	39/94 (41.5%)	0.682
Urine occult blood	8/73 (11.0%)	16/94 (17.0%)	0.268
Urine sugar	3/73 (4.1%)	5/94 (5.3%)	0.717
Female patients			
Urine protein	21/69 (30.4%)	12/48 (25.0%)	0.411
Urine occult blood	19/69 (27.5%)	17/48 (35.4%)	0.364
Urine sugar	1/69 (1.4%)	0/48 (0.0%)	0.402

In both sexes, statistical analyses revealed that the ratios of patients with urine protein, urine occult blood, and urine sugar did not differ significantly between the NF1 patients and controls (Table 2).

### 4.4 Comparison of medical histories between NF1 patients and controls

Patients with NF1 often present with hypertension, renal artery stenosis, pheochromocytoma/paraganglioma, and scoliosis. Gadolinium‐enhanced magnetic resonance imaging (MRI) is sometimes performed on NF1 patients to examine various morphological abnormalities, such as tumours derived from the nervous system and an abnormal skeleton morphology. These factors, which potentially affect renal function, were also examined in the present study. The incidence of gadolinium‐enhanced MRI for male patients and scoliosis for patients of both sexes was significantly higher in the NF1 patients than in the controls (Table S2).

### 4.5 Comparison of renal function between relevant disease‐affected and non‐affected NF1 patients

The effects of medical histories of the comorbidities described above and gadolinium‐enhanced MRI on renal function in NF1 patients were investigated (Tables 3, 4, 5, 6, 7). Creatinine and eGFR were significantly higher and lower, respectively, in male NF1 patients with than in those without a history of hypertension (*p* = 0.002 and *p* < 0.001, respectively; Table 3). eGFR was significantly lower in male NF1 patients with than in those without renal artery stenosis (*p* = 0.026; Table 6). On the other hand, a medical history of gadolinium‐enhanced MRI, pheochromocytoma/paraganglioma, and scoliosis did not correlate with BUN levels, creatinine levels, the BUN/creatinine ratio, or eGFR (Tables 4, 5, and 7).

**TABLE 3:** Comparison of renal function between NF1 patients with and without hypertension

	Hypertension			
	Sex	+	−	*p*‐value
BUN (mg/dl)	Male	16.3 ± 3.7	16.0 ± 9.0	0.290
	Female	15.8 ± 3.9	13.0 ± 3.4	0.022
Creatinine (mg/dl)	Male	0.88 ± 0.12	0.76 ± 0.12	0.002
	Female	0.54 ± 0.11	0.61 ± 0.12	0.950
BUN/Creatinine	Male	18.6 ± 3.5	20.9 ± 9.7	0.520
	Female	30.4 ± 9.6	21.9 ± 7.4	0.092
eGFR (mL/min/1.73m^2^)	Male	71.2 ± 12.3	87.8 ± 14.3	<0.001
	Female	83.6 ± 18.4	92.5 ± 19.2	0.556

*Note*: Mean value and standard deviation are provided.

Abbreviations: BUN, blood urea nitrogen; eGFR, estimated glomerular filtration rate.

**TABLE 4:** Comparison of renal function between NF1 patients with and without gadolinium‐enhanced MRI

	Gadolinium‐enhanced MRI			
	Sex	+	−	*p*‐value
BUN (mg/dl)	Male	15.1 ± 4.2	16.1 ± 8.6	0.880
	Female	14.2 ± 4.9	13.3 ± 3.5	0.474
Creatinine (mg/dl)	Male	0.72 ± 0.10	0.79 ± 0.13	0.122
	Female	0.64 ± 0.14	0.60 ± 0.12	0.763
BUN/Creatinine	Male	20.9 ± 5.1	20.4 ± 9.2	0.478
	Female	22.1 ± 6.5	23.0 ± 8.3	0.597
eGFR (mL/min/1.73m^2^)	Male	88.7 ± 15.1	84.1 ± 15.4	0.367
	Female	80.4 ± 20.4	85.1 ± 18.6	0.975

*Note*: Mean value and standard deviation are provided.

Abbreviations: BUN, blood urea nitrogen; eGFR, estimated glomerular filtration rate; MRI, magnetic resonance imaging.

**TABLE 5:** Comparison of renal function between NF1 patients with and without pheochromocytoma/paraganglioma

	Pheochromocytoma/paraganglioma			
	Sex	+	−	*p*‐value
BUN (mg/dl)	Male	11.0	16.1 ± 8.3	0.286
	Female	na	13.4 ± 3.6	na
Creatinine (mg/dl)	Male	0.69	0.79 ± 0.13	0.468
	Female	na	0.60 ± 0.12	na
BUN/Creatinine	Male	15.9	20.5 ± 8.9	0.519
	Female	na	23.0 ± 8.2	na
eGFR (mL/min/1.73m^2^)	Male	94.8	84.4 ± 15.4	0.494
	Female	na	84.8 ± 18.6	na

*Note*: Mean value and standard deviation are provided. na, not applicable because of the absence of patients with pheochromocytoma/paraganglioma.

Abbreviations: BUN, blood urea nitrogen; eGFR, estimated glomerular filtration rate.

**TABLE 6:** Comparison of renal function between NF1 patients with and without renal artery stenosis

	Renal artery stenosis			
	Sex	+	−	*p*‐value
BUN (mg/dl)	Male	25.0	15.9 ± 8.2	0.780
	Female	na	13.4 ± 3.6	na
Creatinine (mg/dl)	Male	1.10	0.78 ± 0.12	0.052
	Female	na	0.60 ± 0.12	na
BUN/Creatinine	Male	22.7	20.4 ± 8.9	0.519
	Female	na	23.0 ± 8.2	na
eGFR (mL/min/1.73m^2^)	Male	49.4	85.0 ± 14.9	0.026
	Female	na	84.8 ± 18.6	na

*Note*: Mean value and standard deviation are provided. na, not applicable because of the absence of patients with renal artery stenosis.

Abbreviations: BUN, blood urea nitrogen; eGFR, estimated glomerular filtration rate.

**TABLE 7:** Comparison of renal function between NF1 patients with and without scoliosis

	Renal artery stenosis			
	Sex	+	−	*p*‐value
BUN (mg/dl)	Male	14.0 ± 4.6	16.2 ± 8.5	0.317
	Female	13.5 ± 3.5	13.3 ± 3.7	0.756
Creatinine (mg/dl)	Male	0.76 ± 0.09	0.79 ± 0.13	0.732
	Female	0.62 ± 0.15	0.59 ± 0.11	0.643
BUN/Creatinine	Male	18.4 ± 5.4	20.6 ± 9.1	0.718
	Female	22.7 ± 7.1	23.1 ± 8.7	0.345
eGFR (mL/min/1.73m^2^)	Male	89.5 ± 10.2	84.2 ± 15.7	0.314
	Female	82.5 ± 20.6	85.7 ± 17.8	0.764

*Note*: Mean value and standard deviation are provided.

Abbreviations: BUN, blood urea nitrogen; eGFR, estimated glomerular filtration rate.

## 5 DISCUSSION

This study clearly demonstrated that NF1 patients have improved renal function. These results are compelling considering that i) the comparison was performed between age‐ and BMI‐matched NF1 and control patient groups of both sexes, and ii) common results were obtained for both sexes. However, the ratios of patients positive for urine protein or occult blood did not differ significantly between the NF1 patients and controls, a result that contradicts the possibility that clinically overt renal damage occurs less frequently in NF1 patients. In this context, the improved renal function may be a subclinical feature of NF1.

This study showed that the BUN‐to‐creatinine ratio, which is widely used as a marker of acute kidney injury or dehydration,^14^, ^15^ was significantly higher in the NF1 patients than in the controls. Since patients with acute diseases were not eligible for the elective surgery in our hospital, the analysed patient groups naturally did not include patients with acute renal damage. Therefore, possible reasons for the high ratio include: i) NF1 patients may tend to take in relatively large amounts of protein, resulting in high BUN values; ii) NF1 patients may be kept in a protein‐hypercatabolic condition, resulting in high BUN values; iii) NF1 patients may have relatively less muscle mass, resulting in low creatinine levels; and/or iv) NF1 patients may be kept in a dehydrated condition. Considering that some studies have reported that muscle size and strength are decreased in NF1 patients,^16^, ^17^ and that the patients tend to have inadequate intake of various nutrients,^18^ reason i) is unlikely, and reasons ii‐iv) are possible.

The present study showed no significant differences in the prevalence of hypertension, renal artery stenosis, or pheochromocytoma/paraganglioma between the NF1 patients and the controls. On the other hand, Jett et al. reported that the prevalence of hypertension was higher in NF1 patients than in the general population at any age.^19^ Walter et al. also showed that the prevalence of pheochromocytoma/paraganglioma was higher in NF1 patients (5.7%) than in the general population^20^; however, limited information is currently available on the prevalence of renal artery stenosis in NF1 patients. The reason for the lower prevalence of pheochromocytoma/paraganglioma among the patients examined in the present study remains unknown. However, the low prevalence of pheochromocytoma/paraganglioma may be one of the reasons for the lower incidence of hypertension in NF1 patients than in the controls.

Renal function was significantly worse in male NF1 patients with than in those without a history of hypertension. These results demonstrated that hypertension due to any causes, including renal artery stenosis and pheochromocytoma/paraganglioma, impaired renal function, at least in male NF1 patients, which has also been reported for other diseases.^21^ On the other hand, the prevalence of hypertension did not significantly differ between the NF1 patients and controls. Therefore, the prevalence of hypertension does not explain the difference observed in renal function between NF1 patients and the controls.

A medical history of scoliosis did not correlate with renal function in the present study. Riat et al. previously described a 25‐year‐old patient with degenerative lumbar scoliosis leading to severe renal injury through a reduction in the retroperitoneal space around the kidney.^22^ In contrast, Gao et al. reported that routine tests showed normal renal function in children with congenital scoliosis and congenital anomalies of the kidneys and urinary tract.^23^ Therefore, the effects of scoliosis on renal function are controversial. The present results indicated that renal function was superior in NF1 patients than in the controls even though the prevalence of scoliosis was significantly higher in the former than in the latter. Also, no significant difference in renal function was shown between the NF1 patients and controls. Therefore, scoliosis may not significantly affect renal function, at least in the majority of NF1 patients.

The present study demonstrated that a medical history of gadolinium‐enhanced MRI did not correlate with renal function in NF1 patients. Gadolinium does not appear to significantly affect renal function in NF1 patients, although it has been reported to potentially induce nephrogenic systemic fibrosis in patients with impaired renal function.^24^ The present study also showed that a medical history of pheochromocytoma/paraganglioma did not correlate with renal function in the NF1 patients. These results may not be reliable because only 1 patient with pheochromocytoma/paraganglioma was examined. Further studies are needed to elucidate the relationship between renal function and pheochromocytoma/paraganglioma.

Previous literature has suggested a lower prevalence of type 2 diabetes mellitus in NF1 patients.^25^, ^26^, ^27^ These reports are compatible with our data showing that the mean HbA1c level was significantly lower in the NF1 patients than in the controls in both sexes. In that context, NF1 patients may suffer from microangiopathy less frequently, but may have a high risk of macroangiopathy associated with NF1. A low prevalence of renal damage due to microangiopathy may outstrip the risk of renal damage due to macroangiopathy, such as renal artery stenosis, resulting in relatively favourable renal function in the average NF1 patient. More studies are required to confirm this issue.

In this study, renal function was evaluated using the creatinine‐based eGFR. Although peripheral blood creatinine is a marker of renal function, it is not specific for renal function since it is easily affected by other factors, such as age, sex, and muscle burden. Renal function can be examined more precisely by measuring the creatinine clearance, but the process is relatively burdensome, because urine collection is required. The eGFR, which is calculated from the peripheral blood creatinine value, age, and sex, is an easily measured and relatively precise parameter for evaluating renal function, and it is thus widely used for the screening of renal damage.

In this study, the control group consisted of patients with lipoma, atheroma, or melanocytic nevus who were scheduled for surgical excision. The laboratory data from these patients with such localised benign tumours were considered to be suitable as control data since i) the control patients were examined in the same way and with the same analyser as for the NF1 patients, and ii) the non‐NF1 diseases that the control patients had are not associated with renal function. As a consequence, the values of the control patients in this study fell within normal ranges by wide margins.

There are a few limitations in this study. First, data were lacking for smoking and diabetes mellitus; these factors may potentially affect renal function through macro‐ and microangiopathy. Second, an assessment of the clinical severity of NF1 was lacking. Therefore, whether the parameters examined in this study are associated with the clinical severity of NF1 remains unclear. Third, no significant difference was found in the ratio of patients positive for urine sugar between the NF1 patients and control patients, although a significantly lower HbA1c level was shown in the NF1 patients. Considering the low ratio of patients positive for urine sugar in both patient groups, it is possible that the sample number was too small for accurately determining the ratio.

In conclusion, this study demonstrated that NF1 patients have improved renal function. The clinical significances should be further studied.

## CONFLICT OF INTEREST

The authors declare that they have no competing interests.

## ETHICS STATEMENT

The ethics committee of The Jikei University School of Medicine, Tokyo, Japan, approved the study protocol, and informed consent was obtained in the form of opt‐out for all patients.

## AUTHOR CONTRIBUTIONS

Yoshimasa Nobeyama and Ken‐ichi Yasuda analysed and interpreted the patient data. Akihiko Asahina was a major contributor in writing the manuscript. All authors read and approved the final manuscript.

## Supporting information

Supporting Information S1

Supporting Information S2

Supporting Information S3

## Data Availability

Data that support the present results are available in Table S1 and S2.
