# Supplementary material for: Improved renal function in neurofibromatosis type 1 patients
Source: Skin Health Dis. 2022 May 2;2(2):e119. doi: 10.1002/ski2.119 (PMC9168021; doi:10.1002/ski2.119)
Supplement: Supplementary file 3 — Supporting Information S3 [file SKI2-2-e119-s002.doc]

**Supplementary Table 2. Comparison of medical histories between NF1 patients and controls**

No. of affected individuals/ No. of total individuals

Sex NF1 Control *p*-value

Hypertension Male 15/77 (19.5%) 32/102 (31.4%) 0.087

Female 9/71 (12.7%) 6/57 (10.5%) 0.787

Gadolinium-enhanced MRI Male 8/77 (10.4%) 0/102 (0.0%) 0.001

Female 5/71 (7.0%) 0/57 (0.0%) 0.065

Renal artery stenosis Male 1/77 (1.3%) 0/102 (0.0%) 0.430

Female 0/71 (0.0%) 0/57 (0.0%) na

Pheochromocytoma/ paraganglioma Male 1/77 (1.3%) 0/102 (0.0%) 0.430

Female 0/71 (0.0%) 0/57 (0.0%) na

Scoliosis Male 6/77 (7.8%) 1/102 (1.0%) 0.043

Female 21/71 (29.6%) 0/57 (0.0%) <0.001

na, not applicable.
